# Angiocrine endothelium: from physiology to cancer

**DOI:** 10.1186/s12967-020-02244-9

**Published:** 2020-02-03

**Authors:** Jennifer Pasquier, Pegah Ghiabi, Lotfi Chouchane, Kais Razzouk, Shahin Rafii, Arash Rafii

**Affiliations:** 1 Nice Breast Institute, 57 bld de la Californie, 06000 Nice, France; 2 Stem Cell & Microenvironment Laboratory, Weill Cornell Medicine-Qatar, Doha, Qatar; 3 Department of Genetic Medicine, Weill Cornell Medicine, New York, NY 10065 USA; 4 Department of Microbiology and Immunology, Weill Cornell Medicine, New York, NY 10065 USA; 5 Laboratory of Genetic Medicine and Immunology, Weill Cornell Medicine-Qatar, Doha, Qatar

**Keywords:** Angiocrine, Endothelium, Cancer, Cancer microenvironment, Angiogenesis

## Abstract

The concept of cancer as a cell-autonomous disease has been challenged by the wealth of knowledge gathered in the past decades on the importance of tumor microenvironment (TM) in cancer progression and metastasis. The significance of endothelial cells (ECs) in this scenario was initially attributed to their role in vasculogenesis and angiogenesis that is critical for tumor initiation and growth. Nevertheless, the identification of endothelial-derived angiocrine factors illustrated an alternative non-angiogenic function of ECs contributing to both physiological and pathological tissue development. Gene expression profiling studies have demonstrated distinctive expression patterns in tumor-associated endothelial cells that imply a bilateral crosstalk between tumor and its endothelium. Recently, some of the molecular determinants of this reciprocal interaction have been identified which are considered as potential targets for developing novel anti-angiocrine therapeutic strategies.

## Introduction

Metastatic disease accounts for about 90% of patient mortality. The difficulty in controlling and eradicating metastasis might be related to the heterotypic interaction of tumor and its microenvironment [1]. The role of the tumor type specific microenvironment (TM) has been widely described in tumor initiation, maintenance and progression [2–4]. However effective targeting of TM in cancer therapy is not fully appreciated.

The vascular system comprises a vast network of capillary endothelial cells (ECs) that interconnect arteries and veins and create heterogenous vascular beds throughout body. The formation of these vascular networks and their interaction with the surrounding cells is the prerequisite of organ development and tissue regeneration [5, 6]. Similarly, neo-angiogenesis is considered as a critical process for establishing tumor vasculature that ensures tumor survival and expansion. Folkman originally proposed the term “*angiogenic switch*” to imply the essential role of blood vessels in initiation of tumor growth and stated that in the absence of such angiogenesis, tumors cannot expand their mass or display a metastatic phenotype [7]. Based on this theory, many investigators assumed tumor endothelium as the ultimate target for tumor therapy through prevention of neo-vascularization. However, strategies aimed at targeting tumor angiogenesis have mostly failed at clinical trial stage [8], indicating an alternative function for ECs in regulation of tumor progression.

The contribution of tumor endothelium to many hallmarks of cancer has already been reviewed by Hanahan and Coussens [2] and is beyond the scope of this review. In summary, the ECs infer a proliferative advantage to cancer cells in many ways such as neo-angiogenesis and provision of blood supply and nutrients, production of paracrine factors, metabolic regulation, modulation of tumor invasiveness, and resisting cellular apoptosis [5, 9–13]. This review will describe the emerging role of endothelial transmembrane and secretory growth factors and trophogens, referred to as *angiocrine factors*, as essential players in physiological processes and tumor growth [5]. We will describe the concept of angiocrine switch referring to the secreted and membrane bound endothelial factors that participate to the cross talk with cancer cells in association with their role in nutrients and oxygen delivery (angiogenic switch). We will review the data demonstrating that endothelial angiocrine repertoire is heterogenous and organ or tumor type specific. Our aim is to describe the ECs’ angiocrine role in developmental processes and illustrate how these mechanisms are hijacked by tumor cells. We will review the role of endothelial contexture in drug resistance, immune response, and tumor metabolic regulation. Finally, we will discuss new therapeutic approaches disrupting angiocrine endothelium.

### Normal versus tumor endothelium: is there any angiocrine switch?

The importance of angiogenesis in initiation and development of tumors was postulated by Folkman in the context of an angiogenic switch with neovascularization [14]. While tumor vessels displayed an abnormal structure, the existence of a tumor specific endothelium compared to normal endothelium was revealed by gene expression alterations in tumor-associated ECs [15–19]. In addition to genetic alterations, inflammation and hypoxia, in the vicinity of ECs trigger the production of endothelial cytokines promoting tissue repair and tumor growth [20, 21]. Tumor endothelial cells usually display an activated state (discussed below) associated to a change of membrane bound and secretory elements referred here as angiocrine switch. Through the angiocrine switch that occurs early in tumor development, ECs establish a pro-tumoral niche that serves an alternative duty than the previously described angiogenic switch. Hence, the activated tumor ECs play a distinctive role in enhancement of tumorigenicity independent of its function as oxygen and nutrient transporters.

**Fig. 1:**
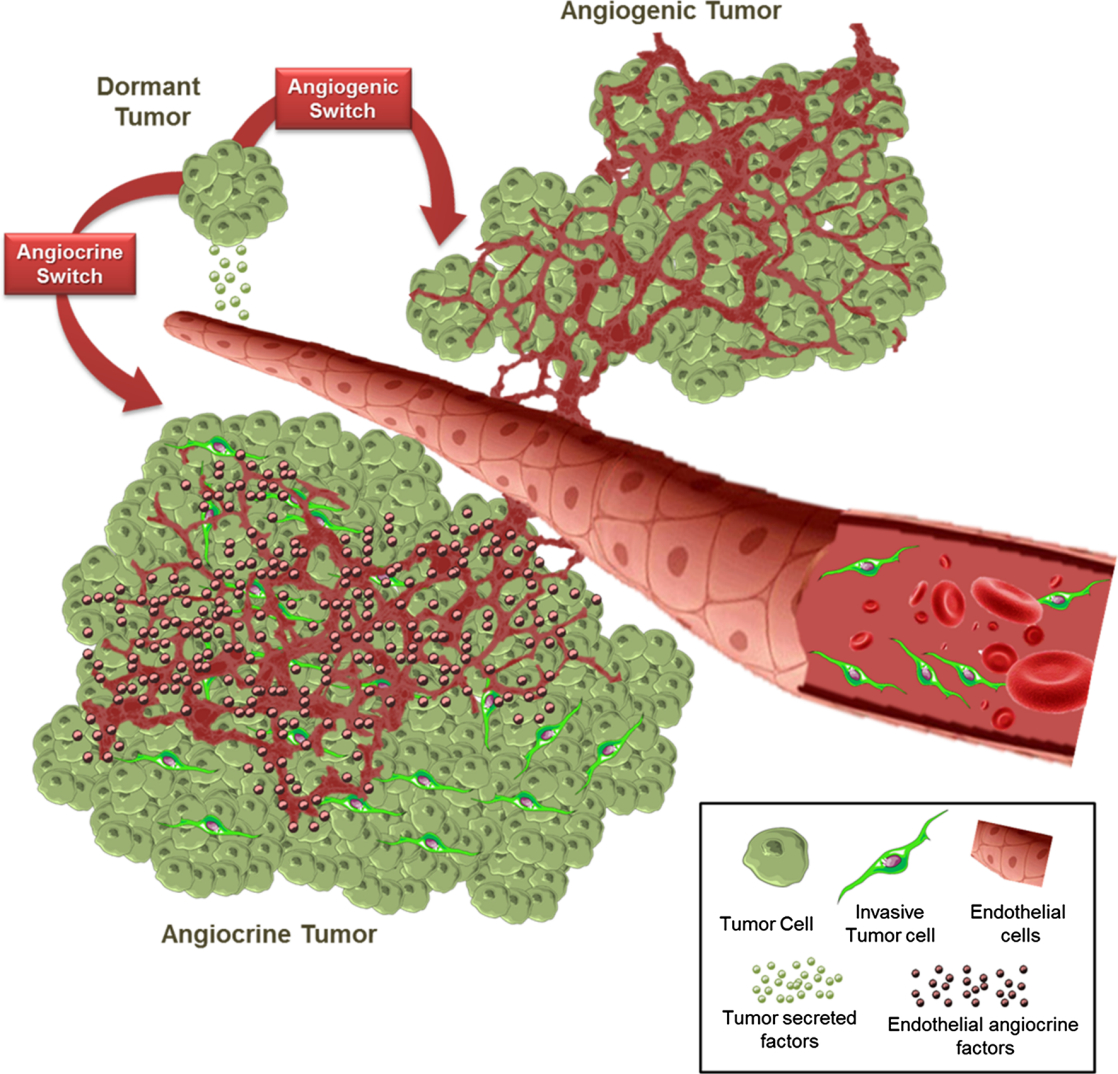
The angiocrine and angiogenic switch during tumor progression. Vascular endothelial cells are capable of enhancing tumor growth and migration by secreting several cytokines and growth factors or activating the so-called angiocrine switch (bottom). This switch is activated by signals directed to ECs from their surrounding tumor cells. As a result, the activated ECs increase the production of angiocrine factors that confer many growth advantages to tumor cells. The angiocrine factors may be in the form of transmembrane ligands, secretory cytokines, or microparticle-packaged factors. There is evidence that activated ECs trough their interaction with tumor cells demonstrate some degree of plasticity toward a mesenchymal phenotype with enhanced tumor promoting potential

In a recent review by Friedl and Alexander, a model has been proposed in which tumor and its microenvironment induces reciprocal changes in their phenotypes and functions (spatiotemporal plasticity) by processing signals they receive from their environment (reciprocity) [22–25]. The interactions occurring in TM are similar to normal tissue development and remodeling processes and mediated by soluble factors or cell-to-cell contacts [3, 4, 26, 27] (Fig. 1). For instance, tumor-derived inflammatory cytokines stimulate the expression of notch ligands Jagged1 and Jagged2 on ECs [28] resulting in notch-dependent tumor growth [29]. In the leukemia model, cancer cell-secreted factors such as VEGF activate ECs in a paracrine fashion to support leukemia expansion by releasing leukemic cell trophogens or by increasing leukemic cell survival through HSP90-mediated induction of Bcl-2 expression and apoptosis inhibition [30–32]. In this model the activation of ECs by neighboring leukemia cells also triggers production of stem cell promoting angiocrine factors such as epidermal growth factors (EGFs) and fibroblast growth factors (FGFs) [33] enriching leukemia stem cells [34]. Similar findings by Cao et al. [35] showed participation of lymphoma-derived FGF4 in priming of a Jagged1 vascular niche that reciprocally reinforced lymphoma propagation and chemoresistance. Jagged1-dependent tumor cells stimulated a transient mesenchymal shift in a subset of tumor ECs in a notch and TGFβ-mediated manner [15]. The mesenchymal ECs (ECs^Mes^) maintain their endothelial identity while acquiring improved angiocrine and angiogenic properties. Although different from endothelial-to-mesenchymal transition (EndMT) phenomenon implicated in the enrichment of cancer-associated fibroblasts (CAFs) [36], this might potentially represent selective activation of tumor endothelium leading to the generation of a pro-tumoral angiocrine endothelium.

### EC angiocrine role in organ development versus tumor progression

**Fig. 2:**
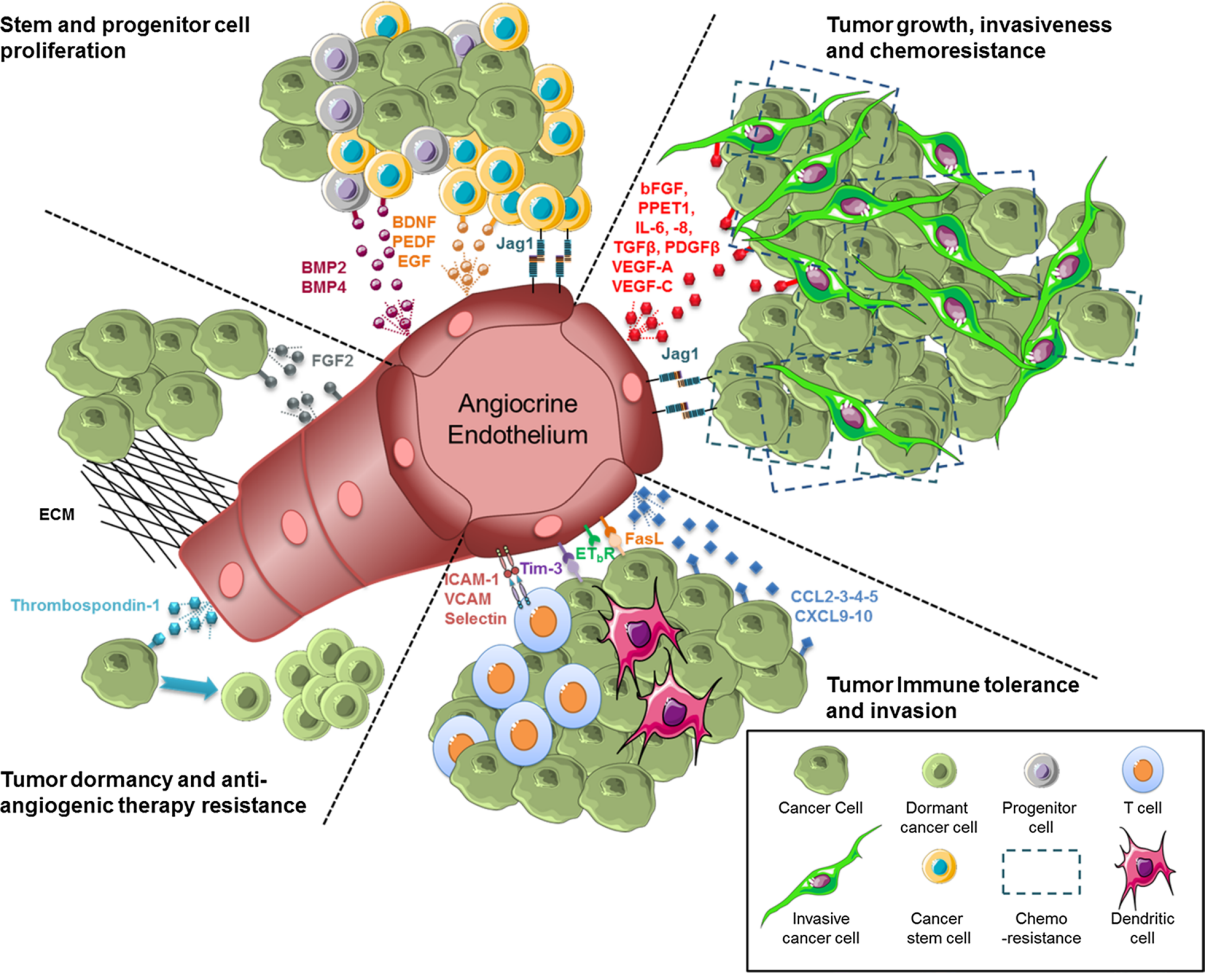
The role of angiocrine endothelium in promoting tumorigenicity. ECs were initially known for their role in tumor angiogenesis. Emerging evidence shows an angiocrine role for tumor endothelium in inducing tumor growth and survival advantages through production of angiocrine factors. Several angiocrine factors such as VEGF, bFGF, IL-6, IL-8, TGFβ, PDGFβ, Jag1, and Endothelin, have been shown to enhance tumor cell proliferation. Also, a role for angiocrine factors is implicated in increased proliferation of cancer stem and progenitor cells in brain tumor (BDNF, PEDF, BMP2, and BMP4), head and neck cancer (EGF), and breast cancer (Jag1). Recent evidence demonstrates the participation of angiocrine endothelium in tumor immune tolerance. Angiocrine molecules such as ICAM-1, VCAM, and Selectin regulate the transmigration of lymphocytes through the lymphatic vessels. Endothelial bFGF plays a role in stimulation of leukocyte infiltration. Some secretory chemokine (CCL2, CCL3, CCL4, CCL5, CXCL9, and CXCL10) affect T cell infiltration to the tumor site. ECs are also capable of over-expressing specific molecules such as ET_B_R to provide a barrier for tumor-infiltrating lymphocytes. Additional mechanisms include escape from immune response regulated by Tim-3 or FasL. A recent angiocrine function for endothelial-derived thrombospondin-1 is defined in induction of tumor dormancy. Besides, endothelial FGF2 is capable of triggering resistance of anti-angiogenic drugs through activation of FGF2/FGFR1 loop. Quite notably, some angiocrine elements may have multiple functions in tumor expansion. Table 1 provides distinct functional information for the viewers

In addition to the primary role of ECs in vasculogenesis and angiogenesis, accumulating evidence shows that ECs contribute to complex patterning occurring during organogenesis [7]. The participation of vascular ECs in organ formation and regeneration has been described in a recent review by our group [6]. The involvement of angiocrine factors in many physiological body processes and their role in stem and progenitor cells regulation suggested their potential involvement in tumor progression and phenotypic plasticity [37–42] (Fig. 2). Here we provide an overview of the ECs angiocrine role in organ development in light of the angiocrine role in tumor progression mediated by secretion/expression of endothelial-specific angiocrine factors described above (Table 1).

**Table 1:** Angiocrine factors involved in tumorigenicity

Angiocrine factors	Function	Tumor specificity	References
BMP2 and BMP4	Control of neural stem/progenitor cell proliferation	Brain tumor	[52]
bFGF, endothelin 1 IL-6, IL-8, TGFβ PDGFβ	Tumor progression	Prostate cancer	[210]
BDNF and PEDF	CSC expansion	Brain tumor	[48, 58]
EGF	CSC expansion	Head and neck cancer	[34]
FasL	Immune tolerance	Ovarian, breast, colon renal, prostate, bladder cancers	[193]
FGF2	Resistance to Bevacizumab in ECs	Ovarian cancer	[150]
Jagged1	Increasing stem cell population Improving tumor growth, stemness and invasiveness Invasiveness and chemoresistance	Colorectal cancer Breast cancer B cell lymphoma	[211] [136] [35]
LAMA4	Generation of vascular niche basement membrane	All types of tumors	[212, 213]
NO	Regulation of leukemic cell growth	Acute Myeloid Leukemia (AML)	[154]
POSTN and TGFβ1	Tumor dormancy	Breast cancer	[144]
CCL2, CCL3, CCL4 CCL5, CXCR9 CXCR10	Tumor immune tolerance	All types of tumors	[190, 214, 215]
VEGF-A and VEGF-C	Improving tumor cell survival	Leukemia	[32, 153]
ICAM1, VCAM Selectin	T cell infiltration into tumors	All types of tumors	[187, 188]
Tim-3	Tumor immune invasion	Lymphoma	[192]

*ANGPT2* angiopoietin 2, *BDNF* brain-derived nerve growth factor, *bFGF* basic fibroblast growth factor, *BMP* bone morphogenetic protein, *CCL* chemokine (c–c motif) ligand (CCL2 also known as MCP1; CCL5 also known as RANTES), *CXCR* chemokine (c-x-c motif) receptor (CXCR9 also known as MIG), *CSF* colony stimulating factor, *EDN1* endothelin 1, *EGF* epidermal growth factor, *FasL* Fas ligand, *FGF2* fibroblast growth factor 2, *ICAM1* intracellular adhesion molecule 1, *IL* interleukin, *LAMA4* laminin α4, *NO* nitric oxide, *PDGFβ* platelet-derived growth factor-β, *PEDF* pigmented epithelial growth factor, *POSTN* periostin (also known as OSF2), *PGF* placental growth factor, *SDF1* stromal cell-derived factor 1 (also known as CXCL2); selectin, also known as CD62, *Tim-3* mucin domain-containing molecule 3, *TGFβ* transforming growth factor-β, *VCAM* vascular cell adhesion molecule, *VEGF* vascular endothelial growth factor

#### Brain

Endothelial cells from dorsal aorta secrete BMPs inducing mesenchymal SDF1 and neuregulin-1 expression, resulting in the attraction of sympatho-adrenal progenitors [43]. BMP signaling from ECs also governs progenitor’s segregation through neuregulin-ErbB signaling, forming the adrenal medulla and sympathetic ganglions. Brain capillaries’ ECs activate quiescent Neural stem cells (NSC) through jagged-1 and EphrinB2 pathways [6]. When activated, NSCs proliferates under the influence of pigment epithelium-derived factor (PEDF) [44], VEGF-C [45], SDF-1 [46] and placental growth factor-2 (PIGF-2) [47] secreted by ECs METTRE LES REF A LA FIN DE LA PHRASE POUR LECTURE PLUS FACILE. Finally, the differentiation of NSCs into neuroblast occurs under the influence of ECs through BDNF [48]. Cleaver et al. [49] have shown a reciprocal paracrine communication between ECs and the neighboring astrocytes mediated by VEGF as an essential factor in tissue differentiation and organ formation. Additional evidence including organization of peripheral nerves along the vascular system, endothelial secretion of neurogenic growth factors like artemin and neurotrophin-3, and neuronal secretion of endothelial stimulating factors illustrate the neuronal-endothelial cross-talk [50, 51]. ECs angiocrine factor have also a role in the regulation of brain stem/progenitor cells physiology. They induce quiescence through activation of BMP/Smad signaling instructing the neural stem cells to exit the cell cycle in the absence of differentiation. They maintain dormancy through EphrinB2 and Jagged-1 expression or stimulation of NO secretion by production of neurotrophin-3 [52–56]. EC in the neurogenic regions of the adult brain stimulate the self-renewal of neurons through PEDF signaling in the adult neural stem cell niche [57, 58]. Endothelial-associated increase in neuronal migration and survival has been attributed to BDNF since blocking this endothelial stimulating factor resulted in complete abrogation of ECs neurotrophic effects [48]. Graded deposition of BDNF and stromal-cell derived factor 1 (SDF-1) by ECs in the mouse brain induce proliferation and migration of brain cells to the olfactory bulb [59].

Brain tumors such as glioblastoma are highly aggressive tumors relying on important angiogenesis. However clinical trials assessing anti-angiogenic therapies such as bevacizumab or sorafenib did not result in the expected results and anti-angiogenic therapies are only considered as second line options. Multiple studies in the literature demonstrate how cancer cells use similar angiocrine cues that during development. Jagged1 ligand through notch activation increases neuroblastoma cells proliferation [60]. In glioblastoma, ECs are able to create a stem cell niche providing NOTCH ligands [61, 62], jagged-1 being essential for the glioma stem cell maintenance and self-renewal [63, 64]. Hence jagged-1 overexpression is linked to poor outcome in [65–67]. EphrinB1 is able to promote oncogenic signaling in medulloblastoma [68]. EphrinB2 expression is associated to the development of glioma and to glioblastoma patient’s prognosis [69]. Endothelial secretion of neuronal growth factors such as VEGF-C, BDNF, BMPs, and PEDF are implicated in brain tumor stem cell enrichment. Neurotrophin signaling through TrkB and TrkC receptors can stimulate brain tumor-initiating cell proliferation [70]. BMP/SMAD pathway is crucial in the development of childhood medulloblastoma [71]. In medulloblastoma, Placental growth factor is responsible for tumor growth and metastasis [72]. Finally the SDF-1/CXCR4 pathway have been widely study in the context of brain tumor (see [73] for review) and is now considered as a potential therapeutic target [74].

#### Liver

The development of the hepatic endoderm is under ECs control though Wnt and Notch signaling. ECs provide critical signals to hepatocytes leading to the establishment of the sinusoidal liver architecture [75] concomittent to biliary differentiation and morphogenesis regulated ECs derived Notch pathway [76]. ECs regulation of liver formation and growth is also mediated through bone morphogenetic protein 4 (BMP-4), fibroblast growth factor 8 (FGF8), and hepatocyte growth factor (HGF) [77–79]. The liver is an organ displaying important plasticity and regenerative ability, Ding et al. demonstrated that a subgroup of liver sinusoidal ECs were able to induce liver regeneration through HGF, Wnt2, and heparin-binding EGF (HB-EGF) activation regardless of liver perfusion [78, 80]. Most importantly EC do play a major role in the balance between fibrosis and regeneration. Fibrosis-free recovery requires CXCR7activation and up-regulation of apelin and follistatin-like-1 angiocrine factors. In contrast chronic liver injury results in activation of CXCR4 and a shift to secretion of angiocrine pro-fibrotic TGFβ1 and BMP2 [81].

In liver cancers (primary hepatocellular carcinoma, cholangiocarcinoma, and fibrolamellar hepatocellular carcinoma) ECs promotes tumor cells through Wnt/β-catenin and Notch pathways activation [82–85]. For example, Notch3 and Notch4 are highly expressed in primary liver cancer while they rarely present in regular liver [86]. Notch4 expression is limited to vascular ECs [87] hence Targeting Notch (especially 3 and 4) signaling could be an anti-angiocrine strategy in liver cancer [88, 89]. ECs derived BMP-4 induce proliferation [90], migration [91] and metastasis [92] in hepatocellular carcinoma and his overexpression is associated with poor prognosis [93]. Secreted FGF8 supports hepatocellular malignancy development and progression during neoangiogenesis [94]. In contact with ECs, cancer cells are stimulated by HGF and overexpress cell adhesion molecules, such as the α2β1 integrin, facilitating migration [95]. CXCR7 and CXCR4 essential for liver regeneration are regulating invasion and tumor growth of human hepatocellular carcinoma cells [96, 97].

#### Lung

The interaction between ECs and epithelial lung cells is essential for lung physiology [98]. ECs secretion of VEGF pathways are primordial in lung morphogenesis and cell proliferation [99]. For example, VEGF and FGF signaling increase metalloproteinase 14 (MMP14) secretion leading to EGF activation in alveolar epithelial cells and bronchioalveolar stem cells inducing their expansion as well as the proliferation of alveolar epithelium [100, 101]. During lung stem cell differentiation, BMP4 production triggers calcineurin/NFATc1-dependent TSP1 expression in ECs, promoting alveolar differentiation and repair [102]. The bilateral crosstalk between pulmonary ECs and lung progenitor cells derives the differentiation of lung epithelial stem and progenitor cells. VEGF, TGF-β1, FGF-2, and PDGF-B secreted by ECs are involved in development and progression of human small cell lung cancer progression and angiogenesis [103, 104]. Consequently, high levels of VEGF and low levels of TSP-1 are associated with a poor prognosis in non-small cell lung cancer [105]. MMP-14 increased expression is associated with poor prognosis in non-small cell lung cancer by increasing metastatic spread [106].

#### Pancreas

Endothelial cells induced endocrine pancreatic differentiation. ECs derived Sphingosine-1-phosphate (S1P) induces budding of pancreatic endoderm which is associated to VEGF pathway activation responsible for the differentiation of insulin-expressing cells [107, 108]. ECs’ laminins interact with β1-integrin and induce both insulin expression and proliferation of beta cells [109]. Kao et al. [110] also demonstrated that ECs play a stage-dependent role in pancreatic development by maintaining pancreatic progenitor cells self-renewal while impairing their differentiation into hormone-expressing cells through secretion of Epidermal growth factor-like domain 7 (EGFL7). Blood-free aortic ECs grown IS1-1-positive mesenchymal cells in vitro were able to selectively influence their differentiation to pancreatic cells thus illustrating the participation of ECs in pancreatic ectoderm differentiation independent of circulating factors [111].

In pancreatic cancer, Sp1 overexpression regulates VEGF expression and angiogenesis [112, 113]. Blockade of Sp1 has demonstrated a significant antitumor activity in pancreatic cancer [114]. Laminins induce pro-survival signal [115]. EGFL7 induce cell invasion and angiogenesis in pancreatic carcinoma and its expression is associated to poor prognosis [116, 117]. The role and expression of VEGF in pancreatic tumor have been widely studied (see [118] for review). VEGF is involved in metastasis process and its expression is associated to higher recurrence rate and poor prognosis [119–121].

#### Kidney

The development of kidney glomeruli is under the close control of ECs. Endothelial VEGF coordinates renal glomerulogenesis and tubulogenesis [122]. While podocytes recruit ECs through VEGF expression and release semaphorins (Sema 3a and Sema 3c) governing kidney vascular morphogenesis [123, 124]. ECs in return secrete MMP2 [125] and platelet-derived growth B (PDGF-B) leading to NNNNNNNNN < [125, 126]. ECs play also a role in renal function through the secretion of glomerular basement membrane formed by extracellular matrix proteins such as Laminin-α5β2γ1 (LM521) forming the glomerular basement membrane [127].

In tumor context, VEGF plays a primordial role in tumor angiogenesis [128]. PDGF-B and VEGF both promotes angiogenesis and cell proliferation in both primary and metastatic clear cell renal cell cancer [129]. MMP2 is involved in invasion and metastasis of clear cell renal cell carcinoma [130, 131]. Laminin-α4 is highly expressed in tumor blood vessels of renal cell carcinoma and is also associated with a poor prognosis [132].

#### Breast

The mammary gland consist of branched tubular structures resulting from the remodeling of epithelial or endothelial sheaths into multicellular tubular networks [133]. Epithelial-to-mesenchymal transition (EMT) is fundamental in breast morphogenesis [134]. ECs contribute to regulation of branching epithelial morphogenesis [135]. The specific pathways implicated in branching morphogenesis and EMT in the breast gland by ECs have not been yet clearly described.

In breast cancer ECs play several roles that have been widely studied from the development of primary tumors to metastatic evolution. ECs increase cancer stem (or initiating) cell (CSC) through contact mediated Jagged1/notch activation [136]. The CSCs participate in cancer progression and recurrence by their increased dormancy, chemoresistance and tumor-initiation potency [137, 138]. In addition, angiocrine-dependent CSCs may contribute to several tumor hallmark capabilities including apoptosis resistance, drug resistance, EMT, and invasion/metastasis [136, 139–141]. We have also gathered evidence on the EC contact- and microparticle-mediated regulation of breast tumor metastasis by showing the up-regulation of EMT markers CA SERAIT BIEN D EN CITER UN PEU in cancer cells once they were pre-exposed to ECs [142]. Our team also demonstrated that the uptake of mitochondria by breast cancer cells from ECs increase their chemoresistance through NNNNNN [143]. Recently Mina Bissel group (A VERIFIER) showed that the secretion of endothelial-derived growth factors influence tumor dormancy [144]. In the context of distant metastasis in the endothelial niche, thrombospondin-1 was shown to induce sustained tumor quiescence, thereby inhibiting tumor expansion and spreading. This suppressive state was inactivated in sprouting neo-vasculature by endothelial-derived tumor-promoting factors such as TGFβ1 and periostin.

#### Ovary

Of all the different organs, the ovary is the organ with the strongest physiological angiogenesis because of its cyclic corpus luteum [145]. Notch based interactions of ovarian cortical and medullar cells with ECs are primordial for ovarian morphogenesis and angiogenesis [146]. ECs in the ovary express Jagged 1, Dll1, and Dll4 ligands while ovarian cells express Notch 1, Notch 2, Notch 3, Notch 4, and Jagged 2. Notch pathway is regulating the VEGF-mediated microvascular growth essential to follicular maturation [147].

In the ovarian cancer, Dll4 and Notch 1 were associated with VEGFR1 and VEGFR2 expression respectively, and impacted microvessel density [148]. When comparing the gene expression in ECs from 10 invasive epithelial ovarian cancers and from five normal ovaries, Lu et al. [16] demonstrated that Jagged 1 was over expressed in invasive epithelial ovarian cancers compared to normal ovary. Dll4, Notch 1, Notch 3 or Jagged 1 are overexpressed in ovarian cancer suggesting that Notch signaling is primordial in ovarian cancer [146]. Finally, we demonstrated the role of angiocrine endothelium in expanding tumor cell population in a contact-dependent as well as microparticle-mediated paracrine fashion [142, 149, 150]. We showed that a co-culture between ovarian cancer cells and ECs induced an increase of ovarian cancer cell proliferation both in vitro and in vivo [151]. This co-culture induced an increase of Notch receptors expression on ovarian cancer cell surface and an increase of Jagged 1 expression on EC surface and activation of survival pathways leading to chemoresistance by ECs.

#### Blood

Hematopoietic stem cell (HSC) give rise to both myeloid and lymphoid lineages. Several lines of evidence confirm a role for angiocrine endothelium in HSC growth and differentiation. (VEGFR-3^+^, VEGFR-2^+^, VE-cadherin^+^, and CD31^+^ Sinusoidal ECs (VEGFR-3^+^, VEGFR-2^+^, VE-cadherin^+^, and CD31^+^) are actively involved in self-renewal, maintenance, and regeneration of HSCs by producing membrane-bound or soluble angiocrine factors such as BMP2, BMP4, insulin growth factor binding protein 2 (IGFBP2), SDF-1, dessert hedgehog (DHH) protein, Notch ligands, wingless-type MMTV integration site 5a (Wnt5a)5a, and kit ligand. Our data shows that angiocrine, but not angiogenic function of ECs determine the lineage-specific differentiation of HSCs through differential secretion of granulocyte macrophage colony-stimulating factor (GM-CSF), interleukin 6 (IL-6), IL-8, granulocyte colony-stimulating factor (G-CSF), IL-1, and tumor necrosis factor (TNF). In Akt-activated endothelium, these angiocrine factors support the expansion of HSCs; whereas co-activation of Akt and p42/44 MAPK in ECs shifts the balance towards maintenance and differentiation of HSCs and progenitor cells [152].

Several reports have suggested the participation of tumor endothelium in neoplastic growth in solid and liquid hematological tumors. Dias et al. [32] demonstrated a non-angiogenic role for VEGF in promoting leukemic cell survival through activation of Hsp90 and induction of Bcl-2 expression. Furthermore, VEGF-C promoted leukemic cell survival and proliferation through interaction with VEGFR-3 (FLT-4) independent of its role in angiogenesis [153]. Other reports showed the angiocrine participation of VEGF signaling in modulation of cell growth in acute myeloid leukemia (AML) by induction of nitric oxide (NO) synthesis [154]. VEGF-mediated angiocrine activation of ECs also plays a role in supporting leukemic progression and resistance to chemotherapy treatment [155]. Inhibition of angiocrine VEGF by blocking VEGFR-2 increase the susceptibility of leukemic cells to chemotherapy. The involvement of vascular ECs in invasiveness and chemoresistance was recently reported in other non-solid hematological tumors [35]. Cao et al. showed that production of FGF4 by B-cell lymphoma and its interaction with FGFR1 receptor upregulates the Notch ligand Jagged 1 on neighboring ECs that reciprocally induces an autocrine loop of Notch2-Hey1 signaling in B cell lymphoma. This crosstalk enforces agressive CD44/IGF1R/CSF1R-positive phenotypes in lymphoma cells with enhanced extranodal invasiveness and chemoresistance.

The complex emerging angiocrine role of ECs may explain the relative failure of many anti-angiogenic therapies. The dichotomous role of ECs in angiogenesis and angiocrine-associated tumor development introduces new therapeutic targets, and instructs us on how to overcome the inefficiency of treatments caused by either redundant or substitutive functions of ECs.

### Angiocrine endothelium and cancer therapy

Currently, cancer is not regarded as a single disease, but as a multi-faceted malignancy manifesting multiple capabilities to sustain proliferation and replicative immortality, evade growth suppression, avoid immune response, inducing angiogenesis, activating invasion and metastasis, promoting genome instability and mutation, resisting apoptosis, and deregulating cellular metabolism [3]. To remain within the scope of this paper, we provide a summary of the main anti-angiogenic strategies used to treat cancer and discuss their advantages and disadvantages. Then, we focus on the newly emerged angiocrine targeting of tumor endothelium and its potential application as adjuvant therapy in combination with other chemotherapeutic agents. The anti-angiogenic therapy originated from the evidence showing the importance of angiogenesis in non-neoplastic diseases as a route for delivery of inflammatory cells to the disease site [156]. The production of pro-angiogenic factors by tumor cells provided additional evidence to propose a role for angiogenesis in cancer development. The importance of VEGF family of pro-angiogenic factors in angiogenesis and tumor neovascularization leaded to the development of several anti-VEGF inhibitors [157–159]. VEGF is secreted by tumor cells and binds to its receptor VEGFR2 and neuropilin on ECs. Bevacizumab (Avastin) is a blocking antibody to VEGF is approved in combination with chemotherapy to treat metastatic colorectal cancer, non-small-cell lung cancer, and breast cancer [160]. Similarly, Ziv-aflibercept (VEGF trap) that functions as a VEGFR decoy is used to treat metastatic colorectal cancer in combination with chemotherapy AUTRE INDICATIONS. Also, an alternative therapy approach benefited from our understanding of the molecular mechanism of vessel stabilization and branching by tumor Angiopoietin1 (Ang-1) and Ang-2 and their tyrosine kinase receptor (Tie-2) on neighboring ECs [161]. Ang-2 antagonizes Ang-1 function in angiogenesis, but while acting in concert with VEGF they can both stabilize and mature new capillaries [162]. Several other angiogenesis inhibitors were approved for cancer therapy (refer to Table 2 for details) that either target receptor tyrosine kinases [163, 164] or reduce VEGF production [165–167]. Although angiogenesis inhibitors display obvious benefits including increased progression-free survival (PFS), the majority of them fail to produce prolonged response impacting overall survival (OS). The transient effectiveness of anti-angiogenesis drugs is reportedly related to two modes of resistance: (1) evasion of anti-angiogenic therapy and (2) indifference to therapy. In an extensive review, Bergers and Hanahan [8] have proposed several strategies that are exploited by each resistance mode including persistence of tumor angiogenesis, tumor blood vessel protection by pericytes, invasion of hypoxic conditions caused by therapeutic agents, and redundancy in angiogenic signals.

**Table 2:** Anti-angiogenesis drugs currently approved for patient use

Drug	Success	Limitation	References
Monoclonal anti-VEGF antibody			
Bevacizumab (Avastin)	Improved PFS in the majority of trials	No OS in the majority of trials	[216–220]
Ramucirumab (Cyramza)	Improved PFS in the majority of trials	No OS in the majority of trials	[221–223]
Chimeric VEGF/PIGF neutralizing receptor			
Ziv-aflibercept (VEGF trap)	Improved PFS in CRC and NSCLC	Little to no OS improvement; no PFS improvement in PACA	[224–226]
Small-molecule VEGFR TKI			
Sorafenib (Nexavar)	Improved PFS in RCC improved OS in HCC	No OS improvement in RCC; no PFS or OS improvement in metastatic melonma or NSCLC	[225, 227, 228]
Sunitinib (Sutent)	Improved PFS in advanced/metastatic RCC, GIST, pancreatic NETs, and PRCA; increased OS in RCC and pancreatic NETs	No OS improvement in the majority of trials, no PFS or OS improvement in metastatic BRCA or CRCA	[225, 229–231]
Pazopanib (Votrient)	Improved PFS in RCC and STS	No OS improvement	[232, 233]
Vandetanib (Caprelsa)	Improved PFS in metastatic MTC	No OS improvement; little/no PFS improvement in metastatic NSCLC	[225, 234, 235]
Vatalanib/PTK787	–	No OS improvement; no/little PFS improvement in metastatic CRCA	[225]
Cediranib	–	No improvement in OS or PFS	[236]
Axitinib (Inlyta)	Improved PFS in RCC	No OS improvement; no PFS improvement in metastatic PACA	[237]
Everolimus (Afinitor)	Improved PFS in BRCA	No OS improvement	[165–167]

*BRCA* breast cancer, *CRC* colorectal cancer, *GIST* gastrointestinal stromal tumor, *HPC* hepatocellular carcinoma, *MTC* medullary thyroid carcinoma, *NET* neuroendocrine tumor, *NSCLC* non-small-cell lung cancer, *OS* overall survival, *PACA* pancreatic cancer, *PFS* progression-free survival, *RCC* renal cell carcinoma

Recent discoveries of angiocrine factors produced by tumor ECs and their critical role in tumor growth and expansion can suggest new targets for drug development (Fig. 2). The angiocrine endothelium could also interfere with anti-angiogenic cancer treatment strategies. A recent work by our group showed a role for Akt-activated angiocrine endothelium in increased resistance to anti-angiogenic drugs such as bevacizumab through stimulation of the autocrine FGF2/FGFR1 loop [150]. The perivascular niche is also implicated in gaining resistance to radiation in medulloblastoma [168]. Hambardzumyan et al. showed that radiation activates the Akt/mTOR pathway in the perivascular nestin-expressing stem cells resulting in their re-entery to cell cycle and restoration of tumor bulk cells leading to tumor recurrence. They further confirmed their finding by demonstrating that inhibition of radiation-induced Akt phosphorylation sensitizes perivascular niche to radiation-induced apoptosis. Similarly the contribution of perivascular niche in promoting chemoresistance has been shown in non-solid tumors. Dias et al. [153] have demonstrated that leukemic cells stimulate ECs through secretion of pro-angiogenic and pro-inflammatory cytokines such as bFGF and IL-1 to VEGF-C interacting with its -3 receptor VEGFR on leukemic cells to induce survival and chemoresistance. Interestingly, Lim et al. [169] showed that inhibition of VEGFR3 on ovarian cancer cells was associated with improved response to chemotherapy through down-regulation of BRCA1 and BRCA2. Cao et al. [35] have also defined a role for vascular niche in inducing chemoresistance in B cell lymphoma. They have demonstrated that interaction of lymphoma cells with tumor ECs results in up-regulation of endothelial Jagged1 and induction of Notch2/Hey1 pathway in lymphoma cells resulting in their improved chemoresistance. Concordant with these findings, our group has shown the participation of tumor endothelial-derived microvesicles in shuttling endothelial angiocrine factors to breast and ovarian cancer cells resulting in enhanced chemoresistance [142, 149]. Taken together, targeting endothelial-specific angiocrine factors might represent several advantages. Targeting those involved in tumor initiation in an adjuvant setting may inhibit tumor recurrence and prevent residual disease; inactivating those that are involved in tumor cell self-renewal and expansion might block tumor growth and metastasis and combinatorial use of those which confer drug resistance might improve chemotherapy outcome.

### Angiocrine endothelium and immune invasion

Most tumor cells express antigens that can mediate recognition by host CD8^+^ T cells. Thus, cancers that are detected clinically must have evaded antitumor immune responses to grow progressively. In solid tumors, it has been shown that the components of TM influence the host immune response (Fig. 2). It has long been recognized that tumor stimulation of an immune response is exceedingly inefficient when tumor is surrounded by microenvironment components. Analysis of the TM in patients with a variety of tumors has revealed that a major subset of tumors shows evidence of a T-cell-infiltrated phenotype. However, the infiltrated T-cells are consequently functionally inhibited [170–172]. The expression of immune checkpoint genes such as programmed cell death 1 ligand 1 (PDL1 or B7-H1), programmed cell death 1 (PD1), and cytotoxic T-lymphocyte-associated protein 4 (CTLA4) in stromal components plays an important role in down-regulation of the immune response to tumor by preventing the activation of T-cells. LaGier et al. have recently shown that the expression of PDL1 on human ECs was associated with reduced T-cell responses, a phenomenon that is also used by tumor cells to escape direct immune destruction [173–175]. Besides, Demaria et al. showed that antitumor efficacy of endothelial-derived interferon response was enhanced by PD1 and CTLA4 blockade [176]. More recently, the checkpoint blockade therapies targeting T-cell inhibitory pathways have produced promising results in many tumor setting [177–180]. Another interesting aspect of stromal regulation of antitumor immune response is associated with the production of angiocrine factors. Some of human tumors that represent poor lymphocyte infiltration appear to have higher expression of several angiogenic factors such as VEGF. VEGF-A that can be produced by both tumor and endothelial cells, is reportedly involved in creation of an immunosuppressive microenvironment through enhancing the production of inhibitory checkpoints molecules such as PD1 [181]. Also, the ineffectiveness of cancer vaccines may be related to the role of tumor endothelium in regulating T-cell homing to tumor location, a process that plays a significant role in optimal anti-tumor immunity and tumor elimination [182–184]. In order for the lymphocytes to enter the TM, they require to transmigrate across lymphatic vessels, a process regulated by endothelial adhesion molecule 1 (ICAM-1), vascular adhesion molecule (VCAM), and Selectin (CD62) as well as local production of specific chemokines [185–187]. Initial report on the participation of tumor endothelium in leukocyte infiltration reported a role for ICAM-1. In their work, Griffioen et al. [188] suggested a regulatory role for angiocrine factor bFGF in stimulating ICAM-1 expression on ECs followed by improved leukocyte infiltration to tumor site. Buckanovich et al. [189] have also shown that the tumor endothelium provides a barrier for tumor-infiltrating lymphocytes by overexpression of endothelin B receptor (ET_B_R). They showed that the overexpression of ET_B_R was correlated with the absence of tumor-infiltrating lymphocytes and poor patient survival. This might be the underlying reason for ineffectiveness of tumor vaccines despite being highly immunogenic. Additionally, Harlin et al. [190] have shown the importance of chemokines such as CCL2, CCL3, CCL4, CCL5, CXCL9, and CXCL10 in T cell infiltration into TM. Although these chemokines are produced by tumor cells, they can also be secreted by stromal cells in the TM and contribute to the recruitment of immune effector cells [191].

As discussed earlier, ECs demonstrate high degrees of plasticity based on their contexture. Emerging data is now revealing the role of tumor cells in activating immune response escape mechanisms in ECs. For instance, the expression of T-cell immunoglobulin and mucin domain-containing molecule 3 (Tim-3) in lymphoma endothelium greatly facilitates the growth and dissemination of lymphoma cells by suppressing CD4^+^ T-cell activation through the stimulation of the IL-6/STAT3 pathway and providing protective immunity [192]. Furthermore, the mechanism by which the tumor vasculature regulates tumor endothelial death barrier and T-cell infiltration in solid tumors has been lately described in establishing immune tolerance [193]. Tumor-derived VEGF-A, IL-10 and prostaglandin E2 were shown to cooperatively induce FasL expression in ECs enabling them to kill effector CD8^+^ T-cells, but not regulatory T-cells.

The above findings highlight the importance of the angiocrine endothelium in regulation of tumor response to immunotherapy and demonstrate that tumor cells are capable of hijacking a physiological anti-inflammatory mechanism of normalizing immune response from angiocrine endothelium to suppress immune reaction against tumor progression.

### The endothelium metabolic regulation of tumor progression

The strong dependency of tumor cells on nutrients and oxygen is the driving force for stimulating blood vessel sprouting. Blood perfusion provides the requirements for cancer progression. Therefore, understanding the connection between tumor nourishment and blood vessel budding may introduce new mechanisms for preventing cancer growth. Recent evidence shows that in addition to tumor-induced angiogenic signals, the EC metabolic requirements are an essential regulators of angiogenesis [194, 195]. Cancer and stromal cells adapt to harsh conditions of TM such as hypoxia and lack of nutrients by switching their metabolism from oxidative phosphorylation to glycolysis [196–198]. The question is when this metabolic conversion happens and how it is regulated? Carmeliet’s group proposed that at the time of vessel sprouting (angiogenic switch), quiescent ECs undergo activation (angiocrine switch) and adapt their metabolism to generate additional energy and biomass for growth and division. This step is referred to as “*metabolic switch*” that occurs in parallel with angiogenic and angiocrine switches [199]. Interestingly, the mechanism of metabolic switch is partly regulated by signals from pro-angiogenic factors produced by tumor cells. De Bock et al. [194, 200] have documented that tumor cells activate the endothelium metabolic switch through VEGF production leading to enhancement of glycolysis by up-regulation of phosphofructokinase-2/fructose-2,6-bisphosphatase 3 (PFKFB3). As a result, a novel hypothesis describes an anti-glycolytic therapy that targets the VEGF/PFKFB3-stimulated glycolysis in vessel sprouting [195]. Inhibition of PFKFB3 resulted in reduced vessel sprouting in endothelial spheroids in zebrafish embryo and postnatal mouse retina as a result of partial and transient reduction in glycolysis. As vessels sprouts by a migratory tip cell and the elongating stalk cells, blocking PFKFB3 will possibly impair the activity of both cell types leading to inhibition of vessel budding. Therefore, inhibition of a key metabolic regulator of ECs might efficiently block neovascularization despite the presence of new pro-angiogenic factors. Furthermore, current anti-angiogenic drugs induce tumor hypoxia and nutrient deprivation that adversely activate the tumor non-oxidative metabolism (glycolysis) contributing to the enrichment of resistant tumor colonies [201]. Hence, identification of angiocrine factors that mediate endothelial metabolism might have implications in developing novel approaches for targeting the concomitant angiocrine, angiogenic, and metabolically activated ECs. Besides, it is possible that anti-metabolism drugs could increase tumor response to therapy by impairing the tumor resistance strategies induced by angiogenic and angiocrine factors. The metabolic regulation of tumor growth introduces a promising area for designing effective anti-metabolism treatments. However, we first need to establish a metabolomic map of the entire metabolites generated in different EC subtypes during different stages of tumor growth and vascular branching to characterize how these compounds are involved in various metabolic pathways [200]. Moreover, tumor and ECs possess some similarities and differences in their metabolic processes. Both cell types are highly glycolytic and use glycolysis for many cellular mechanisms such as proliferation and invasion. These similarities provide great opportunities for targeting both cell types. On the other hand, we must be aware of the differences between tumor and endothelial metabolic needs and mechanisms while applying anti-metabolic strategies. Some of these differences include ability of cancer cells to switch on alternative metabolic pathways, modify their metabolic substrate, and differences in reacting to metabolic stress [202–204].

## Conclusions and future directions

Emerging evidence on the angiocrine role of ECs raises the possibility of designing novel therapy approaches. To ensure the success of anti-angiocrine drugs, precise understanding of all cellular mechanisms involved in the activation of angiocrine switch will be required. This knowledge will be beneficial in designing mechanism-based targeted therapies for interrupting angiocrine activation as well as for impairing the crosstalk between tumor and ECs. While the pathway-specific drugs potentially moderate therapy-induced toxicity, they will provide more effective targeting of endothelial cancer crosstalk. A systematic understanding of the role of angiocrine factors may also lead to functional targeting of ECs. For instance, targeting specific angiocrine factors that foster cancer stem cells may be beneficial in addressing drug resistance and/or tumor recurrence hurdles. However, we ought to learn from earlier angiogenic-targeting therapies and bear in mind that a successful treatment will not be achieved by solely aiming at individual molecules as it may reportedly lead to transient patient survival or development of adaptive patient resistance [2, 205–207]. To avoid succumbing to redundancy in stromal regulation of tumor development, we must first develop comprehensive knowledge of the stroma-tumor interaction to be able to design combinatorial strategies for targeting not only intracellular pathways in cancer cells, but also in cancer stromal cells in many ways [2, 208, 209]. The dichotomous role of ECs in angiogenesis and angiocrine-associated tumor development not only introduces new therapeutic targets, but also instructs us on how to overcome the inefficiency of treatments caused by either redundant or substitutive functions of ECs. This demonstrate that the so called “high jacked” of the surrounding cells by the tumors is in reality the use of regular pathway of the organ by the dedifferentiate cell that composed the tumor. Molecules expressed during embryogenesis and downregulated in adult tissue are re-expressed in tumors.

## Data Availability

Not applicable.

## Abbreviations

AML: Acute myeloid leukemia
bFGF: Basic FGF
BMP-4: Endothelial-derived bone morphogenetic protein 4
CSC: Cancer stem cells
CTLA4T: Lymphocyte-associated protein 4
DHH: Dessert hedgehog
EC: Endothelial Cells
EGFs: Epidermal growth factors
EGFL7: Epidermal growth factor-like domain 7
EMT: Epithelial-to-mesenchymal transition
FDA: Food and Drug Administration
FGFs: Fibroblast growth factors
FGF8: Fibroblast growth factor 8
GM-CSF: Granulocyte macrophage colony-stimulating factor
HGF: Hepatocyte growth factor
HSC: Hematopoietic stem cell
ICAM-1: Endothelial adhesion molecule 1
IGFBP2: Insulin growth factor binding protein 2
IL-6: Interleukin 6
LM521: Laminin-α5β2γ1
MMP14: Matrix metalloproteinase 14
NO: Nitric oxide
NSC: Neural stem cells
OS: Overall survival
PD1: Programmed cell death 1
PDGF-B: Platelet-derived growth B
PDL1: Programmed cell death 1 ligand 1
PFKFB3: Phosphofructokinase-2/fructose-2,6-bisphosphatase 3
PFS: Progression-free survival
SDF-1: Stromal-cell derived factor
S1P: Sphingosine-1-phosphate
TM: Tumor microenvironment
VCAM: Vascular adhesion molecule
